# Future Directions in Diabetic Retinopathy Treatment: Stem Cell Therapy, Nanotechnology, and PPARα Modulation

**DOI:** 10.3390/jcm14030683

**Published:** 2025-01-21

**Authors:** Maria Kąpa, Iga Koryciarz, Natalia Kustosik, Piotr Jurowski, Zofia Pniakowska

**Affiliations:** 1 Department of Ophthalmology and Vision Rehabilitation, Medical University of Lodz, 90-549 Lodz, Poland; maria.kapa@stud.umed.lodz.pl (M.K.); natalia.kustosik@stud.umed.lodz.pl (N.K.); piotr.jurowski@umed.lodz.pl (P.J.); zofia.pniakowska@umed.lodz.pl (Z.P.); 2 Optegra Eye Clinic, 90-127 Lodz, Poland

**Keywords:** diabetic retinopathy, stem cell therapy, diabetes mellitus, nanotechnology, retinal diseases, PPARα, nanoparticles, diabetes, neovascularization

## Abstract

This narrative review focuses on innovative treatment approaches to diabetic retinopathy to meet the urgent demand for advancements in managing both the early and late stages of the disease. Recent studies highlight the potential of adipose stem cells and their secreted factors in mitigating the retinal complications of diabetes, with promising results in improving visual acuity and reducing inflammation and angiogenesis in diabetic retinopathy. However, caution is warranted regarding the safety and long-term therapeutic effects of adipose stem cells transplantation. Bone marrow mesenchymal stem cells can also mitigate retinal damage in diabetic retinopathy. Studies demonstrate that bone marrow mesenchymal stem cells-derived exosomes can suppress the Wnt/β-catenin pathway, reducing oxidative stress, inflammation, and angiogenesis in the diabetic retina, offering promise for future diabetic retinopathy treatments. Nanotechnology has the ability to precisely target the retina and minimize systemic side effects. Nanoparticles and nanocarriers offer improved bioavailability, sustained release of therapeutics, and potential for synergistic effects. They can be a new way of effective treatment and prevention of diabetic retinopathy. Activation and modulation of PPARα as a means for diabetic retinopathy treatment has been widely investigated in recent years and demonstrated promising effects in clinical trials. PPARα activation turned out to be a promising therapeutic method for treating dyslipidemia, inflammation, and insulin sensitivity. The combination of PPARα modulators with small molecules offers an interesting perspective for retinal diseases’ therapy.

## 1. Introduction

Diabetic retinopathy (DR) is one of the most common and severe ocular complications of diabetes mellitus (DM), posing a significant threat to vision [1]. Persistent high blood sugar levels cause damage to the pericytes, leading to leakage of vascular fluid, formation of microaneurysm, and intraretinal hemorrhages [2,3]. DR starts with non-proliferative retinopathy and may progress to proliferative retinopathy if proper diagnosis and treatment are not implemented [4]. Proliferative DR can be a direct cause of visual acuity deterioration and in advanced stages may lead to irreversible vision loss [5]. The global prevalence of diabetes continues to rise, as does the incidence of DR. It has become one of the major public health concerns [6].

DR occurs in approximately 77.3% of patients with type 1 diabetes (T1D) and 25.1% of those with type 2 diabetes (T2D). Among these individuals, around 25–30% are at risk of developing vision-threatening complications, such as diabetic macular edema (DME), which further exacerbates the risk of severe vision loss [1].

According to the International Diabetes Federation, approximately 537 million adults worldwide were living with diabetes in 2021, and this number is expected to increase to 783 million by 2045. Among these individuals, an estimated one-third are affected by some form of DR [6].

Globally, DR is a leading cause of blindness in working-age adults [2]. The prevalence of DR among diabetic patients has risen to 22.27%, making it the fifth leading cause of blindness worldwide. However, this burden varies significantly by region, with rates reaching 17% in North America and Australia, 15–17% in Europe, and ranging from 3 to 7% in other parts of the world [7,8].

Currently, the role of DR prevention is being emphasized as the best method to maintain good quality of vision and, consequently, to improve the quality of life of diabetic patients. The risk factors for DR confirmed by evidence-based medicine include duration of diabetes, unregulated or poorly treated diabetes, high blood glucose levels, high glycated hemoglobin levels (HbA1C), hypertension, hyperlipidemia, abnormal cholesterol levels, pregnancy, and smoking [9]. Educating patients about their ability to influence modifiable risk factors for diabetes, such as blood sugar and cholesterol levels, blood pressure regulation, regular HBA1C monitoring, as well as their lifestyle: a healthy diet, physical activity, and regular eye check-ups, is the basis for achieving good results in the treatment of DR [10].

Current treatment options for DR include laser therapy, intravitreal injections of anti-VEGF drugs, and corticosteroids, which aim to slow disease progression and prevent vision loss [8]. However, these treatments mostly target advanced stages of the disease, when significant damage has already occurred. There is a requirement for effective and safe modern therapies focused on both early and late stages of DR [11]. Traditional therapies have been widely applied to the clinical prevention and treatment of DR, delivering satisfactory efficacy in inhibiting retinal neovascularization, relieving retinal macular edema, and removing the stretching of fibrous tissues during the proliferation period for the retina [12]. Nevertheless, these treatments are often invasive, require repeated interventions, and may not fully address the underlying damage to the retina. As a result, there is a growing interest in exploring new, more effective therapies that can target the disease at earlier stages and improve patient outcomes.

In this narrative review, we would like to concentrate on selected forms of future treatment of DR, such as stem cell therapy and the use of nanoparticles (NPs), which are promising frontiers in the treatment of DR.

## 2. Stem Cell Therapy in DR

The popularity of stem cell therapy (SCT) is steadily increasing, indicating a growing trend within this domain. Stem cells (SCs) exhibit abilities to undergo differentiation into diverse cell types, rendering them versatile and applicable across various fields of medicine. Over the past few decades, significant research efforts have been dedicated to exploring the therapeutic potential of stem cells in tissue regeneration and repair. Stem cells can be derived from a variety of tissues, including bone marrow, adipose tissue, amniotic cells, umbilical cord, and placental tissue [13]. Each source presents unique advantages and challenges, particularly concerning factors such as immunogenicity and therapeutic efficacy.

This section aims to provide a comprehensive review of the current state of stem cell therapy in the treatment of diabetic retinopathy. It will critically examine recent research findings, therapeutic outcomes, and the diverse stem cell sources being explored for their potential to mitigate the progression of DR. By synthesizing current evidence and discussing emerging trends, this chapter will highlight the evolving role of SCT in DR management, while also addressing the challenges and future research directions that could further advance the clinical applicability of SCT for retinal diseases.

### 2.1. Adipose Stem Cells

Adipose stem cells (ASCs) are classified as multipotent mesenchymal stem cells, exhibiting the capability to differentiate into a varied amount of tissue cells [14]. Numerous reports show that ASCs have the ability to enhance insulin sensitivity and mitigate the number of dead β-cells. Moreover, ASCs have immunomodulatory attributes, which can regulate metabolic inflammation during various metabolic diseases such as DM. This promises not only the potential for halting the progression of DR but also for preventing its development entirely [15,16].

The study conducted on the Ins2Akita mice has shown that ASCs or their secreted factors lessen retinal complications of diabetes. Researchers injected intravitreal CD140b-positive ASCs (1000 cells/1 μL) or 20× conditioned media from cytokine-primed ASCs (ASC-CM, 1 μL). The control group of mice received saline, subsequently. After 3 weeks, visual function experiments and histological analyses were made. Ins2Akita mice that received ASC-CM had better visual acuity and no adverse complications in comparison to CD140b-positive ASCs [17,18,19].

As shown in recent studies, the coexistence of inflammation, endothelial cell proliferation, and neovascularization has a huge impact on the pathogenesis of DR [20,21]. The research investigated the potential role of extracellular vesicles (EVs) derived from adipose mesenchymal stem cells (MSCs) in angiogenesis and endothelial proliferation in DR. Researchers focused specifically on the function of microRNA-192 (miR-192) in delaying DR by targeting overexpressed integrin subunit α1 (ITGA1). Diabetes in rats was induced by streptozotocin (STZ). The study demonstrated that reducing the expression of ITGA1 alleviated inflammation and angiogenesis in the diabetic retina induced by STZ. Additionally, the researchers observed that MSC-derived EVs mitigated the inflammatory response and angiogenesis by transporting miR-192, which targeted ITGA1, resulting in its negative response and leading to ameliorating diabetic retinal damage. The findings indicated that miR-192, released by EVs from MSCs, played a crucial role in delaying the events of inflammation and angiogenesis in DR [22].

The results obtained in another study showed that adipose tissue-sourced mesenchymal stem cells-derived exosomes (AT-MSC-Exos) prevented apoptotic cell death and reduced inflammation in both neural and retinal tissues. Future investigations should prioritize the development of novel genetic strategies to augment the efficiency of AT-MSC-Exos as drug delivery in the fields of regenerative neurology and ophthalmology [23].

Researchers investigated the efficacy of ASC transplantation in retinal degenerative diseases (RDD) using a rat model (the 6-week-old Sprague-Dawley rats). They compared outcomes between intravitreal and subretinal transplantation of rat ASCs. Intravitreal transplantation led to reduced neural retinal damage but extinguished electric response and membrane formation, subretinal transplantation resulted in partial recovery of electric response and reduced rosette formation. The study suggests that the subretinal space may be a more effective site for ASC-based therapy in RDD treatment [24].

Intravitreal injections of ASCs made at a stem cell clinic in the United States led to serious adverse events in three patients. The study investigates the significant bilateral visual loss related to these injections. Prior to the injections, the patients exhibited varying degrees of visual acuity, ranging from 20/30 to 20/200. However, post-injection, they suffered from severe visual impairment accompanied by ocular hypertension, hemorrhagic retinopathy, vitreous hemorrhage, combined traction, and rhegmatogenous retinal detachment or lens dislocation. After a year, their visual acuity deteriorated considerably, with measurements ranging from 20/200 to complete loss of light perception. These cases emphasize the urgent need for thorough scrutiny and regulation of stem cell clinics to prevent such devastating outcomes and protect patients’ ocular health studies shed new light on the therapeutic mechanisms of ASCs [25]. Nonetheless, further investigation should be conducted to confirm whether ASCs or their products are safe and have long-term therapeutic effects.

### 2.2. Bone Marrow Mesenchymal Stem Cells

Bone marrow mesenchymal stem cells (BM-MSCs) exhibit a distinctive feature comparing to other somatic stem cells: they possess the ability to differentiate into various cell types beyond the typical mesodermal-lineage, including ectodermal and endodermal lineages. This unique versatility makes BM-MSCs a distinct category within adult stem cells [26]. In this review we would like to summarize and present some of the newest data with regard to this issue.

The research made on rats which had diabetes induced by STZ, aimed to assess the potential effectiveness of exosomes derived from bone marrow mesenchymal stem cells (BM-MSCs-Ex) in mitigating retinal damage induced by targeting the Wnt/β-catenin signaling pathway. Findings revealed that these exosomes effectively inhibited the Wnt/β-catenin pathway in the diabetic retina, resulting in notable reductions in oxidative stress markers, increased expression of antioxidant enzymes, and decreased levels of inflammatory and angiogenic factors. These results were further supported by histopathological analyses, fundus examinations, and optical coherence tomography scans. Overall, this study demonstrates that MSC-derived exosomes alleviate diabetes-induced retinal damage by suppressing the Wnt/β-catenin pathway leading to a reduction in oxidative stress, inflammation, and angiogenesis [27].

In the subsequent research, the investigators endeavored to determine if the protective attributes of CD34+ Bone Marrow Stromal Cells (BMSCs) could be observed in all three layers of the capillary plexus of the retina after intravitreal administration. The results showed that intravitreal injection of human CD34+ BMSCs led to significantly higher vascular density in the superficial retinal capillary plexus layer in a murine model of DR. These findings suggest these injections may protect the retinal vasculature in diabetic eyes. Future clinical trials may explore the use of intravitreal CD34+ BMSC injections for treating vision loss in DR [28].

The major challenge in achieving successful subretinal transplantation of MSCs for retinal degeneration remains their limited survival. Researchers investigated the potential of IL-13 (interleukin-13) gene modification to improve the survival of MSCs transplanted into the subretinal space. To conduct this study, lipopolysaccharides-activated retinal microglia (RMG) were cocultured with MSCs or MSCs engineered to express IL-13 for 24 h. Activated phenotypes were detected in vitro, where, compared to normal MSCs, IL-13-expressing MSCs effectively reduced the expression of pro-inflammatory markers and major histocompatibility complex II. These results indicate that IL-13-MSCs have the ability to shift activated RMG towards a neuroprotective M2 phenotype, which not only enhances the survival of transplanted MSCs after subretinal transplantation but also suggests a potential role in mitigating graft rejection. To investigate further, the survival of graft MSCs was quantitatively evaluated in vivo, showing that more transplanted cells survived in the IL-13-MSCs group than in the MSCs group six weeks following subretinal transplantation [29].

As shown in the previous study, SCT encounters numerous limitations and difficulties. MSCs are promising for clinical use due to their potential for allogeneic transplantation and immune-modulatory effects. However, their complex immunomodulatory effects, including potential proinflammatory responses, raise concerns about their safety and efficacy, particularly in intravitreal applications for conditions like DR. In studies using non-obese diabetic-severe combined immune deficiency (NOD-SCID) mice, intravitreal injection of MSCs led to the formation of tractional epiretinal membrane, raising concerns about their clinical use in the eye [30].

Furthermore, MSCs exhibit both pro- and anti-tumorigenic properties depending on various complex factors, such as type of tissue, secretome, interactions with cancer and host immune cells, type of cancer, and the specific conditions of the study (in vivo or in vitro). MSCs can promote tumors through cell–cell fusion with cancer cells, creating hybrids with cancer stem cell traits. On the other hand, MSCs may also suppress tumors by triggering inflammatory cell infiltration, inhibiting angiogenesis, arresting the cell cycle, inducing apoptosis, and blocking survival and migration pathways like Wnt/β-catenin [31].

In the prospective, nonrandomized, phase I, open-label study—conducted on three eyes of three volunteers with advanced retinitis pigmentosa—tumor formation was assessed by transplanting patient-derived MSCs into the retinas and subcutaneous regions of B6Nude mice (n = 6). The results showed no tumor formation within six months after treatment, suggesting a degree of safety under these conditions [32].

However, the safety of intravitreal MSC implantation remains controversial. A phase I clinical trial reported that intravitreal injection of autologous bone marrow MSCs did not meet safety standards, with significant side effects including fibrosis and tractional retinal detachment. Furthermore, allogeneic MSC transplantation into the vitreous chamber was shown to induce severe inflammation and retinal damage, leading to loss of both visual and retinal function [33].

In this comprehensive review, we focused on ASCs, BM-MSCs, and perinatal stem cells, however, there are other stem cells used in DR, such as embryonic stem cells [34] or pluripotent stem cells [35]. SCT shows promise in treating DR, with ASCs offering the potential to stop disease progression. Recent studies highlight ASCs’ ability to enhance insulin sensitivity, regulate inflammation, and aid in retinal tissue regeneration [36]. EVs from ASCs also show effectiveness in reducing inflammation and angiogenesis in DR [37]. However, caution is needed due to adverse events observed in patients receiving intravitreal ASC injections. Additionally, BM-MSCs show the potential to mitigate retinal damage by targeting harmful signaling pathways [38]. Nevertheless, limited survival and immune-related concerns need to be taken into consideration when applied in DR.

### 2.3. Perinatal Stem Cells

#### 2.3.1. Amniotic Stem Cells

Amniotic stem cells are derived from amniotic fluid or amniotic membrane (the innermost layer of two membranes surrounding the fetus), which provides an environment for the developing fetus. These cells combine properties of both embryonic and adult stem cells, offering the ability to differentiate into multiple tissue types such as neural tissue, neuroectodermal tissue, bone, cartilage, and fat. Their anti-inflammatory properties and regenerative potential make them promising candidates for treating conditions like neurodegenerative diseases, cardiovascular issues, and tissue injuries. Amniotic stem cells are an exciting focus in regenerative medicine, given their versatility and accessibility [39,40].

Amniotic membrane stem cells (AMSCs) are promising for DR treatment due to the exosome-rich conditioned medium (ERCM) derived from them. In the study conducted in 2023 [41], 28 male Sprague-Dawley rats were divided into 3 groups: a normal control group, a DM group, and a DM group treated with ERCM (DM-ERCM). DM was induced via intraperitoneal streptozotocin injection, and the DM-ERCM group received subconjunctival injections of ERCM containing 1.2 × 10⁹ exosomes, administered four times every 2 weeks. Results demonstrated that subconjunctival ERCM significantly improved retinal function in diabetic rats, with higher b-wave and flicker amplitudes observed on electroretinography and reduced severity of retinal vascular attenuation and retinal layer damage. Despite these promising findings, the study was limited by its small sample size and reliance on an animal model [41].

Building on the potential of amniotic stem cells for retinal disease treatment, another study explored the use of human amniotic fluid-derived mesenchymal stromal cells (hAFSCs) and recombinant human Nerve Growth Factor (rhNGF) for addressing DR-associated neurodegeneration. Researchers utilized porcine neuroretinal explants exposed to high glucose (25 mM) to simulate DR and bioengineered decellularized human corneal lenticules (hCLs) with hAFSCs, rhNGF-loaded microparticles, or both. Co-culture of bioengineered hCLs with high-glucose-treated neuroretinal explants reduced markers of inflammation, oxidative stress, apoptosis, and angiogenesis while enhancing retinal cell markers. While the results demonstrate the feasibility and therapeutic promise of hCLs as an ocular drug delivery system, limitations include the need for in vivo studies to validate these findings and assess long-term safety and effectiveness in clinical settings [42].

According to these works, further research is essential to confirm the long-term safety, effectiveness, and feasibility of translating amniotic stem cell therapies to human clinical applications.

#### 2.3.2. Placental Stem Cells

Similarly to amniotic stem cells, placental stem cells offer a versatile and ethically favorable source for regenerative therapies, with unique properties that expand their potential clinical applications. They are pluripotent cells derived from the placenta, offering the ability to differentiate into all three embryonic germ layers. They are non-carcinogenic and ethically uncontroversial, as fetal membranes are discarded after birth. Due to these advantages, they hold promise for a wide range of clinical applications, including in the treatment of neurological, liver, heart, and other diseases [43].

In the study conducted on 22 Lewis rats, researchers investigated human placental stem cells for their potential to treat DR by intravitreal injection [44]. Rat models were treated with an intravitreal microinjection of hMSCs derived from the placenta. The release of neurotrophic factors from stem cells in the vitreous cavity was measured using real-time polymerase chain reaction. Results showed detectable levels of various neurotrophic factors, including basic fibroblast growth factor, nerve growth factor, and brain-derived neurotrophic factor, all of which were significantly higher in the vitreous of treated rats compared to control groups. The rats receiving the stem cell injection demonstrated improved fluorangiographic outcomes, with reduced hypofluorescence and ischemia, indicating slowed or stabilized DR progression [44].

#### 2.3.3. Umbilical Cord Stem Cells

Human umbilical cord mesenchymal stem cells (HUCMSCs) are multipotent cells derived from the umbilical cord, including compartments such as Wharton’s jelly, cord lining, and perivascular tissue. These ethically noncontroversial stem cells can differentiate into the three germ layers, making them a valuable source for regenerative medicine, immune modulation, and cancer therapy [45].

The first study aimed to investigate whether HUCMSCs-derived exosomes carrying miR-17-3p could ameliorate DR by targeting the STAT1 gene. A mouse model of DM was established, and HUCMSC-derived exosomes containing elevated miR-17-3p were injected into these mice. Researchers measured parameters such as blood glucose, glycosylated hemoglobin, inflammatory markers, oxidative stress factors, and retinal cell apoptosis to assess the therapeutic effects of the miR-17-3p-enriched exosomes. Results showed that the exosomes improved metabolic indicators, reduced inflammation and oxidative injury, and decreased apoptosis in the retinal tissues of the diabetic mice. However, the study’s limitations include the relatively short observation period and reliance on an animal model, which necessitate further investigation to validate these findings in a clinical [46].

Another study aimed to evaluate the effects of intravitreal injection of neural stem cells (NSCs), originating from HUCMSCs, on neurodegeneration in a rat model of DR. In this approach, HUCMSCs were first isolated and cultured, then induced to differentiate into NSCs using a neural differentiation medium. Four weeks after transplantation, rats exhibited reduced retinal vascular dysfunction, elevated levels of brain-derived neurotrophic factor and Thy-1 compared to non-treated controls. Moreover, the morphological improvements coincided with vision restoration, as shown by the flash electroretinograph. Notably, NSCs derived from HUCMSCs boosted survival rates of retinal ganglion cells and mitigated the progression of DR, highlighting their neuroprotective potential [47].

These findings suggest that the usage of HUCMSCs might be a promising therapeutic strategy for addressing neurodegeneration in DR.

## 3. Nanotechnology in DR

Nanotechnology has emerged as a transformative approach to addressing the significant challenges associated with drug delivery for DR. The anatomical and physiological barriers of the eye, including the tear film, cornea, conjunctiva, sclera, the blood–aqueous barrier (BAB), and the blood–retinal barrier (BRB), impose substantial limitations on the delivery of therapeutic agents to the posterior segment, where the retinal tissues reside [12,48]. Conventional ocular drug delivery methods, such as eye drops and ointments, are predominantly effective for anterior segment diseases, with less than 0.001% of the administered drug penetrating intraocular tissues [49]. In contrast, nanotechnology-based drug delivery systems, particularly nanoparticles (NPs), have demonstrated remarkable potential in overcoming these barriers. With a size range of 1 to 1000 nanometres, which results in a high surface area-to-volume ratio, nanoparticles possess unique physicochemical properties that enhance drug solubility, protect therapeutic agents from enzymatic degradation, and enable controlled, sustained drug release [12]. These attributes contribute to improved ocular bioavailability, prolonged therapeutic effects, and reduced dosing frequency, ultimately enhancing treatment outcomes and patient compliance.

Topical application of nanoparticle-based formulations provides a non-invasive strategy for drug delivery to the posterior segment. Nanoparticles composed of materials such as chitosan, hyaluronic acid, and alginate demonstrate mucoadhesive properties, enabling them to adhere to the ocular surface, resist rapid clearance by tear fluid, and prolong contact with the corneal and conjunctival epithelium [50]. These characteristics facilitate enhanced drug permeation across ocular barriers, allowing efficient drug transport to the retina without requiring invasive procedures. Surface modifications can further enhance nanoparticle efficacy. For example, nanoparticles with neutral or slightly positive surface charges exhibit improved ocular penetration [51]. Additionally, functionalization with targeting ligands, such as antibodies or peptides, enables specific interactions with retinal cells, such as the retinal pigment epithelium (RPE), improving drug localization and minimizing systemic side effects [49].

Recent studies highlight the potential of surface-modified nanoparticles for non-invasive retinal drug delivery. Silva et al. (2023) demonstrated that chitosan–hyaluronic acid nanoparticles effectively delivered epoetin beta (EPOβ) to the retina of Wistar Hannover rats via topical administration, with the drug detectable in retinal tissues for up to 21 days [52]. Similarly, Laddha et al. (2022) developed PLGA nanoparticles loaded with curcumin for the topical treatment of DR showing that these nanoparticles reduced VEGF levels in the vitreous fluid of rats, confirming their potential for targeted retinal therapies [53]. Both studies underscore the ability of nanoparticle-based formulations to provide controlled, long-term, and targeted treatments for retinal diseases, offering significant benefits over traditional invasive therapies while enhancing patient compliance [52,53].

Nanoparticles offer several advantages over traditional ocular drug delivery systems. By encapsulating therapeutic agents, they protect drugs from premature degradation and enable sustained release, reducing administration frequency and improving overall treatment efficacy [49]. Furthermore, nanoparticles can encapsulate multiple therapeutic agents within a single carrier, facilitating synergistic effects while minimizing systemic toxicity. Emerging nanomaterials, such as graphene, also hold promise in retinal therapies, with graphene’s electrical conductivity supporting retinal ganglion cell survival and promoting axonal regeneration, indicating its potential beyond drug delivery alone [12].

As nanotechnology continues to evolve, new materials and approaches are emerging. In the following sections, we will explore various types of nanocarriers, such as lipid nanoparticles, nanoliposomes, polymeric nanoparticles, dendrimers, and others that have shown considerable potential in overcoming barriers to effective retinal drug delivery, further enhancing the treatment of DR and related conditions.

### 3.1. Lipids

**Figure 1:**
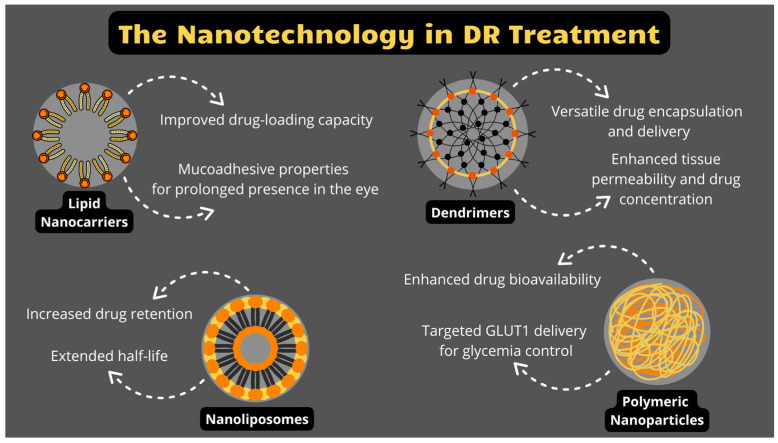
Schematic summary of molecules used in nanotechnology in DR treatment; pt. 1.

Lipid NPs, including solid lipid NPs (SLNPs) and nanostructured lipid carriers (NLCs), possess a lipid core with dimensions within the nanometre range, stabilized by a surfactant layer. Notably, both lipophilic and hydrophilic drugs can be loaded into such a lipid core and NLCs are able to accommodate both liquid and solid lipids, making them advantageous compared to SLNPs. Their benefits include increased drug-loading capacity, improved stability, and controlled drug release. Additionally, the mucoadhesive properties of lipid NPs enhance their interface with ocular mucosa, prolonging drug presence in the eye (Figure 1). Overall, lipid NPs exhibit several advantages, such as colloidal carriers, including high drug loading rates, long-term stability, and minimal biotoxicity [54,55,56]. In a study conducted on diabetic rats, chitosan-modified 5-Fluorouracil NLCs (CS-5-FU-NLCs) prepared by modified melt emulsification-ultrasonication method showed improved drug release and anti-angiogenic effects in vitro and ex vivo compared to a traditional 5-FU solution, proving that it is possible to deliver topical medications deep into the posterior segment of the eye [57]. Abd El hakim Ramadan et al. developed optimized SLNPs containing vildagliptin (VLD), which they inserted into Ocuserts’ (OCUs) matrices (VLD-SLNPs-OCUs) for easier and safer administration to the eye and to avoid the typical side effects of orally administered VLD, such as upper respiratory tract infection, nausea, diarrhea and hypoglycemia. VLD aids retinal blood flow and reduces ocular inflammation in patients with T2D and VLD-SLNPs-OCUs offer the possibility to use its benefits with fewer systemic side effects, longer retention, extended release, and more stable plasma concentrations [58].

### 3.2. Nanoliposomes

Nanoliposomes are minute vesicles composed of a single or multiple lipid bilayers encapsulating an aqueous core, which serve as versatile drug-delivery vehicles for both lipophilic and hydrophilic drugs [59]. Drug-loaded nanoliposomes exhibit enhanced biocompatibility and smaller size compared to standalone drugs, facilitating prolonged drug retention, extended half-life and interaction with the target (Figure 1) [60]. A study involving intravenous administration of cyclosporin A-loaded lipid nanocapsules to DR mice demonstrated a disruption to the inflammatory cycle with reduced microglial activation, macrophage recruitment, and inhibition of pro-inflammatory cytokine release [61]. Nanoliposomes show promising potential in treating retinal conditions. However, they are not without their drawbacks; they can cause blurry vision, trigger inflammation during cationic delivery, and tend to aggregate in vivo due to sub-optimal colloidal stability [48].

### 3.3. Polymeric Nanoparticles

Polymeric NPs are synthesized using a diverse range of biocompatible natural and synthetic polymers [62]. Among the latter, polylactide (PLA) and poly-lactic-co-glycolic acid (PLGA) are commonly employed in the formulation of polymeric NPs. Additionally, natural compounds such as chitosan [63], hyaluronic acid [64], and cyclodextrin [65] have been utilized in their preparation, facilitating an effective drug delivery to the posterior component of the eye.

The mucoadhesive polymer, chitosan (CS), enhances the pre-ocular drug presence through an interaction of its positively charged amino groups with the negatively charged sialic acid residues in the mucosa [56].

It has been shown that retinal capillaries in diabetes are characterized by decreased expression of the glucose transporter 1 (GLUT1), resulting in accumulation of glucose in retina, inducing the development of DR (Figure 1) [58,59,60,61,62,63,64,65,66]. Experimental results showed that the subretinal delivery of GLUT1 via hyaluronic acid coated NCs, targeting the CD44 receptor of RPE cells, can aid in the control of glycemia in diabetic retinas in mice. What is more, these NPs exhibited neuroprotective effect on the optic nerve and were generally safe and non-toxic to the retina [12].

Triamcinolone acetonide (TA) is an FDA-approved, long-acting glucocorticosteroid widely used in DR treatment for its anti-inflammatory properties. Despite its hydrophobicity and low molecular weight, its effectiveness at the target side leaves much to be desired. Various NCs, including PLGA-NPs, polymeric NPs, dendrimers and micro-implants, have been tested to enhance the delivery of TA [67]. NPs especially improve the drug’s durability (T1/2) and concentration (Cmax), bioavailability and release, while simultaneously decreasing toxicity and dosing frequency [68]. Incorporating TA into SLNs and in situ gel formulations resulted in enhanced pre-corneal residence time and sustained drug delivery to both the anterior and posterior segment ocular tissues [69]. Dandamudi and colleagues formulated CS-coated PLGA NPs loaded with TA for topical ocular administration in the treatment of acquired retinal vasculopathies [70]. The mucoadhesive properties of CS can also be synergistically combined with hyaluronic acid for further enhanced performance and efficacy [71].

Jeong et al. investigated the application of human serum albumin (HSA) as a reservoir for transportation of the water-insoluble apatinib (Apa)—a novel and selective VEGF receptor 2 inhibitor. Researchers encapsulated the drug within NPs composed of HSA-conjugated polyethylene glycol (PEG). In vitro, these Apa-HSA-PEG NPs demonstrated significant inhibition of VEGF-induced endothelial hyperpermeability in human retinal microvascular endothelial cells. When administered via intravitreal injection in mice, they successfully blocked VEGF-induced retinal vascular leakage and more importantly, substantially curtailed diabetes-induced vascular leakage in STZ-induced diabetic mice [72].

### 3.4. Dendrimers

Dendrimers have garnered significant interest as nanostructured polymers due to their versatility and multi-branched structure, resembling a tree [73]. The functional groups at the end of their branches may be neutral, negatively or positively charged. Drugs can be covalently conjugated or entrapped within the dendrimer network through ionic interactions, hydrophobic interactions, or hydrogen bonds. Dendrimers based on poly-amidoamine (PAMAM) polymers have received considerable attention in drug delivery due to their ease of manufacturing [74]. Dexamethasone-PAMAM conjugates administered via a subconjunctival injection demonstrated enhanced ocular tissue permeability and concentration of the steroid drug (Figure 1) [48].

### 3.5. Peptides

**Figure 2:**
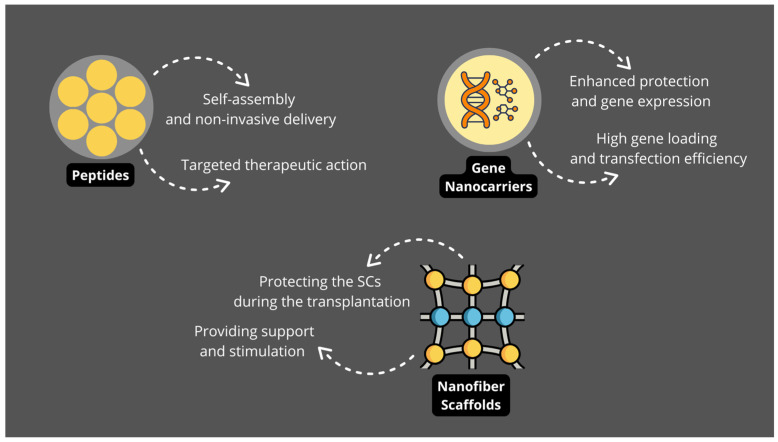
Schematic summary of molecules used in nanotechnology in DR treatment; pt. 2.

With their multitude of structural and biological roles, peptides have become widely utilized in disease therapeutics. A recent study focused on developing a smart supramolecular peptide (SSP) eye drop tailored for DR treatment, targeting the sSema 4D/PlexinB1 signaling specifically. This pathway has been shown to enable pathological neovascularization and leakage in diabetic retinas, through influences on endothelial cell migration, proliferation and phosphorylation and pericyte internalization. The SSP eye drops successfully reach the retina and vitreous humor, where they can precisely target and, upon self-assembling and forming a fibrous network, effectively capture the sSema 4D. As a result, sSema 4D is unable to interact with its three receptors, which warrants a significant reduction in pathologic neovascularization in the retina. Importantly, this study proves that such non-invasive methods can provide therapeutic effects on par with invasive antibody injections and, when combined with anti-VEGF treatment, these SSP eye drops enhanced the current clinical efficacy by 50% (Figure 2) [75].

### 3.6. Treatment Monitoring

Nanotechnology-facilitated drug delivery offers not only a smoother passage through the BRB and a better durability of the substance, but also the possibility for close drug monitoring. Upon coating two anti-VEGF medications, bevacizumab and aflibercept, with carbon nanodots (C-dots), Shoval et al. observed that the fluorescence of the C-dots allowed for non-invasive monitoring of the intraocular drug concentration. Additionally, the envelopes were preventing the degradation of the drugs before they entered the retina, thus leading to increased concentration and effectiveness of the therapeutics [76].

### 3.7. Nanotechnology for Retinal Regeneration

Retinal neurodegeneration, a hallmark of DR and other retinal disorders, is thought to arise primarily from an imbalance of neurotrophic factors, oxidative stress, and chronic inflammation, which lead to nerve damage and apoptosis. Nanomaterials offer promising solutions for addressing these challenges by promoting retinal tissue repair and regeneration. Electrospun and self-assembled nanofibers are among the most widely utilized, fabricated from natural polymers (fibrin, gelatine, laminin, collagen) and synthetic (PCL, PLGA, PLA) fibers. These nanofibers are primarily administered via localized implantation or topical application to the ocular surface, targeting retinal neurodegeneration and promoting regeneration of damaged nerve tissues. For instance, Chang et al. developed an electrospun multichannel nerve guidance nanofiber conduit loaded with nerve growth factor (NGF) for immediate release to induce early axon regeneration and brain-derived neurotrophic factor (BDGF) for sustained release to support axonal growth and myelin sheath formation. Their study demonstrated that the conduit significantly enhanced nerve regeneration and functional recovery in preclinical models of nerve injury, a mechanism highly relevant for retinal pathologies requiring nerve repair [77]. Additionally, nanofiber scaffolds, such as those incorporating PCL and PLGA, have shown the ability to mimic the extracellular matrix, supporting cell adhesion, proliferation, and differentiation. For example, Thompson et al. utilized two-photon polymerized polycaprolactone (PCL) scaffolds to enhance retinal cell attachment and survival in models of retinal degeneration, demonstrating their biocompatibility and potential for retinal tissue engineering [78]. However, further studies are needed to optimize these nanofiber designs and assess their long-term efficacy and biocompatibility in models of diabetic retinopathy and related retinal disorders.

Furthermore, SC-based therapies combined with nanotechnology seem particularly promising for restoration of damaged retinal tissue. This combination provides a solution to a common limitation of SCT, which is cell death upon injection, before the needed functional integration with retinal tissue can occur. Natural nanofiber scaffolds may solve this issue by protecting the SCs from mechanical and oxidative stress during and after the transplantation and providing structural and biochemical cues that promote cell viability, differentiation, and integration with the retinal microenvironment. Soleimannejad et al. demonstrated that CJMSCs embedded in fibrin hydrogel exhibited improved viability and a higher expression of photoreceptor-specific markers, such as rhodopsin, protein kinase C (PKC), recoverin, and peripherin, compared to cells cultured on standard tissue culture plates. The study utilized an ex vivo retinal explant model to mimic retinal microenvironments and pathologies. The fibrin encapsulation was found to enhance photoreceptor differentiation by providing a three-dimensional (3D) matrix that mimics the native extracellular matrix (ECM), thus promising a therapeutic advantage. Moreover, the scaffold facilitated robust attachment of CJMSCs and supported their interaction with the surrounding tissue without inducing cytotoxic effects. This biocompatibility and functionality underscore the potential of fibrin hydrogels as scaffolds in retinal regeneration strategies [79].

### 3.8. Gene Nanocarriers

In addition to nano-scaffolds, nanomaterials also serve as efficient gene delivery devices for cell reprogramming in ocular regeneration, particularly in DR. One prominent non-viral gene nanocarrier (NC) is the liposome–protamine–DNA (LPD) complex. These complexes protect plasmid DNA (typically 5–10 kb in size) from enzymatic degradation and enhance transfection efficiency [80]. The inclusion of cholesterol as a helper lipid enhances in vivo transfection efficiency, reducing the need for cationic lipids [81].

Notably, LPD nanoparticles have been investigated for gene delivery to retinal ganglion cells via intravitreal injection, achieving cell-specific expression of reporter genes and demonstrating potential for targeted ocular therapy (Figure 2) [82].

Another promising approach is the use of liposome–polyethylenimine (LPP) complexes, which have been utilized to deliver HuR siRNA (~21 nucleotides) intravitreally in streptozotocin-induced diabetic rats. This treatment effectively decreased retinal VEGF levels and mitigated retinopathy progression, highlighting the ability of LPP complexes to address key pathological processes in DR [83]. These studies suggest that non-viral liposome-based nanocarriers offer versatile platforms for delivering therapeutic genes, ranging from small siRNAs to larger plasmid DNAs, thereby reducing pathological angiogenesis and improving retinal health [83].

Organic–inorganic hybrid nanocrystals represent another type of gene NC with significant potential. These hybrid nanocarriers enhanced gene transfection and transgene expression by 20- and 3-fold, respectively, compared to non-modified inorganic carbonate apatite nanocrystals and the commercially available Lipofectamine. These results, obtained in embryonic mouse cells, demonstrate the great promise of hybrid NCs for stem cell reprogramming (Figure 2) [84].

The nanocarrier systems discussed above have demonstrated promising results in in vivo DR models, with intravitreal administration ensuring localized delivery to retinal tissues. Transfection durations typically range from 24 to 72 h, with outcomes such as enhanced gene expression, reduced VEGF-mediated angiogenesis, and mitigation of retinal pathology. These advances underscore the therapeutic potential of nanocarriers for gene delivery in diabetic retinopathy and other retinal diseases.

### 3.9. Limitations of Nanocarriers

In the field of drug-loaded NCs, both researchers and regulatory agencies grapple with multifaceted challenges, which necessitate robust characterization techniques, scalable optimization strategies, rigorous safety protocols, and stability preservation. Each phase of a nanocarrier’s path to clinical use is marked by logistical and technical hurdles [54]. Physiological factors—such as temperature, pH, and ionic strength—exert a significant influence on NCs’ physicochemical properties, including charge and hydrodynamic diameter [85]. Post-injection, NCs’ binding to plasma proteins can alter their distribution, clearance, and drug release profile in physiological fluids. Additionally, unpredictable changes to substance toxicity and biocompatibility may occur upon interactions with bodily fluids, which lead to modifications in polydispersity. Ensuring the safety of NCs in drug delivery systems (DDSs) remains a paramount challenge and factors like size, shape, surface charge, delivery route, and drug dosage all play a role in determining NCs’ potential toxicity [54].

A comprehensive characterization of NCs in vivo requires cytotoxicity studies, which can be successfully and cost-effectively conducted in cell culture models for acute nanotoxicity [86,87]. However, in those models, low cellular availability hinders the investigation of long-exposure or repeated-exposure toxicity, two areas in which the research is still visibly lacking. Additionally, nanocarrier systems based on peptides and nucleic acids may trigger immunogenic reactions with significant adverse effects, including anaphylactic shock [54].

Oversight of nanocarrier usage falls under the purview of regulatory bodies such as the FDA, the European Medicines Agency (EMA), and the Center for Drug Evaluation and Research (CDER) [88]. Large-scale nanocarrier production necessitates vigilant monitoring of exposure levels and potential effects to establish a favorable safety profile. While novel toxicological techniques, like particokinetics and multiparametric analysis have been explored, there is no globally accepted standardized approach for assessing nanocarrier toxicity. This coupled with no well-defined guidelines for characterization and quality control makes the transition from laboratory settings to clinical trials an arduous task. Considering safety implications, factors such as size, surface charge, and solubility can be leveraged to predict nanocarrier toxicity, as recommended by international standard-setting organizations. Given the limited historical acceptance in the scientific literature and the fact that only 21 nanocarrier compositions have secured regulatory bodies approval in the last 3 decades, there is a pressing need to establish and validate novel standardized techniques for nanocarrier safety and characterization [54].

## 4. PPARα Receptors in DR Therapy

The progression of DR is affected by blood glucose control, arterial blood pressure, and cholesterol levels [89]. While DR is a major cause of blindness in the developed world, current treatments are limited to invasive procedures suitable only for late stages and can have significant side effects [90,91]. Notably, over 40% of DR patients fail to respond to the anti-VEGF treatment, which represents the gold standard in DR therapy [92]. There is a pressing need for early-stage, patient-friendly treatments. Oral PPARα agonists are emerging as a non-invasive preventive treatment option [93].

PPARα, part of a family of nuclear receptors, is involved in lipid and carbohydrate metabolism [94] and is widely expressed in the body [95]. Activation of PPARα has been demonstrated to elevate plasma high-density lipoprotein cholesterol levels while concurrently reducing triglycerides, free fatty acids, and apolipoprotein concentrations, thereby ameliorating the lipid profile and exerting beneficial effects on inflammatory processes and insulin sensitivity [96,97]. The growing body of evidence positions PPARα activation as a viable therapeutic target for a spectrum of pathologies, including cardiovascular diseases [98], dyslipidaemia [99], diabetes, and its associated sequelae, notably DR [96].

Research indicates that PPARα are expressed in various tissues affected by the microvascular diabetic diseases—the retina, kidney and nerves [100,101], with notably reduced expression in diabetic retinas. Previous studies demonstrated more prominent retinal acellular capillary formation, pericyte dropout and exacerbated retinal neurodegeneration in PPARα−/− mice with diabetes compared to diabetic wild-type mice [102,103]. In diabetic retinas an increase of oxidative stress markers—Gstm1 (glutathione-s-transferase m1), Prdx6 (peroxidase 6) and Txnrd1 (thioredoxin reductase 1)—was further enhanced with lack of PPARα [103].

### 4.1. Fenofibrate-PPARα Agonist

A well-established PPARα agonist—fenofibrate has been commonly prescribed for hyperlipidaemia, but researchers have explored its therapeutic potential beyond lipid modulation, especially in prevention of DR [104,105]. Notably, fenofibrate treatment has been associated with the inhibition of vascular endothelial growth factor C (VEGFC) and vascular endothelial growth factor receptor-3 (VEGFR-3) expression in human RPE cells under hypoxic conditions, which is the driving force behind choroidal neovascularization—hallmark of DR progression [106].

Clinical studies have shed light on fenofibrate’s impact on DR. The fenofibrate intervention and event lowering in diabetes (FIELD) study revealed that fenofibrate use could reduce the need for initial laser photocoagulation in individuals with pre-existing retinopathy [107]. Similarly, the action to control cardiovascular risk in diabetes (ACCORD) study, while not showing a statistically significant difference in moderate vision loss between placebo and fenofibrate-administered groups, did observe a slower DR progression in the latter group [108].

Beyond these findings, additional therapeutic effects of fenofibrate have been explored, such as its influence on endothelial progenitor and circulating progenitor cell levels in DR patients [93]. A 12-week parallel-group RCT comparing fenofibrate vs. placebo in 41 patients with DR showed significant increase in circulating hematopoietic stem/progenitor cells (HSPCs) in the fenofibrate group, which corresponded to more favorable retinopathy outcomes. Furthermore, endothelial differentiation of CD34+ cells was significantly reduced by fenofibrate [109].

Oxidative stress is another one of key factors in diabetes-associated retinal neurodegeneration. In 4-hydroxynonenal-induced oxidative stress conditions, fenofibric acid (active metabolite of fenofibrate) in mice reduced mitochondrial oxidative stress and cell death in cultured retinal neuronal cells, suggesting a possible reversibility of mitochondrial dysfunction caused by oxidative stress in DR retinas [103].

Experimental findings in animals indicate that fenofibric acid may be successful in protecting against DR, as it reduced ganglion cells death and managed to conserve oscillatory potentials of b-wave in diabetic mice after oral administration [110]. Furthermore, the heightened expression of inflammatory and apoptotic markers, such as IL-6, IL-1beta, P53, Bax and VEGF was reduced in diabetic retinas of rats upon oral dosing, with similar effects found in human retinal capillary endothelial cells (RCECs) [111].

However, caution is warranted in clinical practice. Fenofibrate’s primary renal excretion pathway poses risks for patients with impaired kidney function. Therefore, it is not highly recommended for individuals with severe renal diseases to undergo fenofibrate therapy. As research continues, the balance between therapeutic benefits and potential adverse effects remains a critical consideration in managing DR [112,113].

### 4.2. Pemafibrate-Selective PPARα Modulator (SPPARMα)

Pemafibrate, a novel selective PPARα modulator, exhibits greater efficacy and specificity in activating PPARα compared to fenofibrate [114,115,116]. It is associated with enhanced triglyceride reduction, increased high-density lipoprotein cholesterol, and fewer renal side effects [117]. Structural distinctions to fenofibrate, such as the addition of a phenoxy alkyl group (group B) and the 2-aminobenzoxazolic group (Group C), improve its binding affinity to PPARα (via hydrophobic interactions and Group B flexibility), leading to more precise activation [118,119]. Fewer adverse effects on kidney function can be attributed to predominantly hepatic metabolism and biliary excretion, as opposed to other fibrates.

In experimental models, pemafibrate has demonstrated retinal protective effects. For instance, in oxygen-induced retinopathy (OIR) models, oral administration of pemafibrate increased plasma fibroblast growth factor 21 (FGF21) levels, inhibited HIF-1α and VEGFA expressions, and reduced retinal neovascularization [120]. It also aids in improving blood glucose levels and decreasing serum levels of insulin [120,121]. Moreover, pemafibrate also preserved retinal function in streptozotocin (STZ)-induced diabetic mouse models by maintaining synaptophysin expression, a key regulator of synaptic vesicle endocytosis [120,122]. Finally, it could potentially increase thrombomodulin expression in RCECs, aiding a faster regeneration of the retina through suppression of vascular leakage, leukostasis and inflammation [123].

While fenofibrate has demonstrated efficacy in reducing the need for laser photocoagulation and slowing DR progression in large clinical trials such as FIELD and ACCORD [107,108], pemafibrate’s pharmacological profile offers advantages in terms of safety and lipid-modulating efficacy. However, it is important to note that direct comparative studies between pemafibrate and fenofibrate in DR patients are currently limited. Moreover, unlike fenofibrate, which has direct retinal effects, some of pemafibrate’s benefits appear to be mediated through systemic mechanisms, such as liver-specific PPARα activation [120]. Thus, while pemafibrate shows promise as a therapeutic option, further studies are necessary to fully evaluate its comparative efficacy and long-term benefits in DR management.

### 4.3. Y-0452-PPARα Agonist

In 2017, Ma Lab at the University of Oklahoma Health Science Center reported a new PPARα agonist, 7-chloro-8-methyl-2-phenylquinoline-4-carboxylic acid (Y-0452), experimentally demonstrated to exhibit anti-apoptotic and neuroprotective effects in R28 (photoreceptor precursors’ derived cell line) and inhibit angiogenesis in human RCECs. Moreover, this agonistic chemotype contributes to a significant decrease in retinal inflammation and apoptosis, with no observed toxicity in mice and diabetic rats. However, Y-0452 displays weak on-target activity in biochemical PPARα assays (EC50 ≈ 25–50 µM) and a low-level agonism compared to known agonists [124]. The highly functionalized quinoline core of the compound was suspected to affect the ligand–protein interaction, ultimately decreasing target engagement [125].

### 4.4. A91 and A190-PPARα Agonists

Based on the challenges observed in the structure–activity relationship of Y-0452, scientists hypothesized that transposing the carboxylic acid and deconstructing the quinoline system might improve the selectivity and compatibility of the receptor’s binding pocket. Hence, A91, a novel class 4-benzyloxy-benzylamino PPARα agonist was identified, showing a promising EC50 = ~4 µM and manifesting >20-fold selectivity for PPARα over the γ and δ isoforms [126], as well as an advanced analogue—A190, showing a ~100-fold better EC50 of ~40 nM in the cell-based luciferase assay and a >2700-fold PPARα isoform selectivity [127]. Research demonstrated that both chemotypes are effective in vivo for treating STZ-induced DR in rats after systemic administration. They exhibit good bioavailability, maintain their efficacy after first-pass metabolism, and present a low safety risk with no observed toxicity after a month of daily treatments. Further improvements to this therapeutic approach may bring its potency and selectivity on par with pemafibrate [125].

## 5. Conclusions

In recent years, DR management has advanced significantly, leveraging SCT, nanotechnology, and PPARα modulation to address the disease’s underlying mechanisms while mitigating the limitations of existing treatments.

SCT holds transformative potential, with ASCs and BM-MSCs leading the charge. ASCs have been shown to enhance insulin sensitivity and reduce retinal inflammation through the secretion of extracellular vesicles carrying microRNA-192, which targets pro-inflammatory pathways like ITGA1. BM-MSC-derived exosomes, meanwhile, effectively mitigate oxidative stress and angiogenesis by suppressing the Wnt/β-catenin signaling pathway. Perinatal stem cells, such as amniotic and umbilical cord stem cells, also exhibit significant regenerative capacity, including the promotion of neuroprotection via exosome-rich conditioned media and nerve growth factor delivery. However, challenges such as variability in cell survival rates, immune rejection risks, tumorigenic effects, and ad-verse outcomes like fibrosis and retinal detachment persist. Prioritizing standardized protocols, improved cell-sourcing methods, and advanced delivery systems, such as nanoscaffolds, is essential for enhancing safety and efficacy.

Nanotechnology has emerged as a transformative approach to ocular drug delivery, overcoming the eye’s anatomical and physiological barriers, such as the blood–retinal barrier, to deliver therapies more efficiently and with fewer systemic side effects. Lipid nanoparticles and polymeric nanocarriers, such as chitosan–hyaluronic acid nanoparticles, have demonstrated success in delivering drugs like epoetin beta and curcumin to retinal tissues, achieving sustained release and reducing VEGF levels. Additionally, dendrimers and nanofiber scaffolds provide structural and biochemical cues for retinal repair, mimicking the extracellular matrix to support photoreceptor regeneration. However, the potential risks of nanotechnology remain a concern, including nanotoxicity, immune reactions, and difficulties in large-scale manufacturing. Addressing these challenges requires comprehensive toxicological evaluations, particularly for nanomaterials that remain in ocular tissues for extended periods. Advances in biodegradable nanomaterials and targeted delivery mechanisms are promising areas for further investigation. Regulatory frameworks must also be adapted to ensure the safety and scalability of nanotechnology for widespread clinical use.

Parallel to these advancements, PPARα modulation has shown promising results in managing DR, particularly in its ability to modulate lipid profiles, reduce oxidative stress, and promote neuroprotection. Fenofibrate and pemafibrate, two PPARα agonists, have demonstrated efficacy in slowing DR progression and reducing the need for laser photocoagulation, a standard but invasive treatment for advanced DR. Pemafibrate, with its im-proved safety profile and more targeted lipid modulation, presents a potentially more favorable option compared to fenofibrate, particularly in reducing renal side effects. Additionally, emerging PPARα agonists, such as Y-0452 and newer compounds like A91 and A190, have demonstrated anti-apoptotic, neuroprotective, and anti-inflammatory effects in preclinical studies, showing great potential for protecting retinal neurons from damage in the early stages of DR. Nonetheless, clinical translation of these agents requires careful evaluation of their safety profiles, particularly for patients with renal or hepatic impairments. Comparative studies are needed to clarify the relative advantages of newer modulators and optimize dosing strategies for long-term use.

The integration of SCT, nanotechnology, and PPARα modulation offers promising advancements in DR management. Combining SCT with nanotechnology-based scaffolds could improve cell survival, while PPARα modulators may enhance retinal repair. However, further exploration is needed to maximize their synergy, address challenges like immune compatibility, tumorigenicity, and potential toxicity, and ensure patient accessibility and cost-effectiveness. Long-term clinical trials are essential to assess the safety and efficacy of these therapies, particularly in patients with complex comorbidities. Ethical considerations and collaboration among researchers, clinicians, and policymakers will be crucial for overcoming hurdles and translating these innovations into clinical practice. Interdisciplinary research holds the potential to revolutionize DR management, offering new strategies to slow or even reverse disease progression, improving the quality of life for millions worldwide.

## Data Availability

No new data were created or analyzed in this study. Data sharing is not applicable to this article.

## Abbreviations

5-FU	5-Fluorouracil
ACCORD	Action to control cardiovascular risk in diabetes
AMSCs	Amniotic membrane stem cells
ASCs	Adipose stem cells
ASC-CM	Adipose-derived stem cell conditioned medium
AT-MSC-Exos	Adipose tissue-sourced mesenchymal stem cells-derived exosomes
BAB	Blood–aqueous barrier
BDGF	Brain-derived growth factor
BM-MSC	Bone marrow mesenchymal stem cells
BMSCs	Bone marrow stem cells
BRB	Blood–retina barrier
C-dots	Carbon nanodots
CDER	Center for Drug Evaluation and Research
CJMSCs	Conjunctival mesenchymal stem cells
CS	Mucoadhesive polymer chitosan
CS-5-FU-NLCs	Chitosan-modified 5-Fluorouracil nanostructured lipid carriers
DDSs	Drug delivery system
DM-ERCM	DM group treated with ERCM
DME	Diabetic macular edema
DR	Diabetic retinopathy
EMA	European Medicines Agency
ERCM	Exosome-rich conditioned medium
EVs	Extracellular vesicles
FIELD	Fenofibrate intervention and event lowering in diabetes
FGF21	Fibroblast growth factor
GLUT1	Glucose transporter 1
HAS	Human serum albumin
HBA1C	Glycated hemoglobin
hAFSCs	Human amniotic fluid-derived mesenchymal stromal cells
hCLs	Human corneal lenticules
HSPCs	Hematopoietic stem/progenitor cells
HUCMSCs	Human umbilical cord mesenchymal stem cells
IL-13	Interleukin-13
ITGA1	Integrin subunit α1
LPD	Liposome-protamine-DNA
miRNA-192	MicroRNA-192
MSCs	Mesenchymal stem cells
NCs	Nanocarriers
NLCs	Nanostructured lipid carriers
NGF	Nerve growth factor
NOD-SCID	Non-obese diabetic-severe combined immune deficiency
NPs	Nanoparticles
NSCs	Neural stem cells
OIR	Oxygen -induced retinopathy
PAMAM	Poly-amidoamine
PEG	Polyethylene glycol
PLA	Polylactide
PLGA	Poly-lactic-co-glycolic acid
RCECs	Retinal capillary endothelial cells
RDD	Retinal degenerative diseases
RGCs	Retinal ganglionic cells
rhNGF	Recombinant human nerve growth factor
RMG	Retinal microglia
RPE	Retinal pigment epithelium
SCs	Stem cells
SCT	Stem cell therapy
SLNPs	Solid lipid nanoparticles
SPPARMα	Selective PPARα modulator
SSP	Smart supramolecular peptide
STZ	Streptozotocin
TA	Triamcinolone acetonide
T1D	Type 1 diabetes
T2D	Type 2 diabetes
VEGFC	Vascular endothelial growth factor
VEGFR-3	Vascular endothelial growth factor receptor-3
Y-0452	7-chloro-8-methyl-2-phenylquinoline-4-carboxylic acid
